# Enhancing compliance with waste sorting regulations through inspections and motivational interviewing

**DOI:** 10.1177/0734242X231154145

**Published:** 2023-02-26

**Authors:** Mathias Herzing, Hans Wickström, Adam Jacobsson, Håkan Källmén, Lars Forsberg

**Affiliations:** 1 Department of Economics, Stockholm University, Stockholm, Sweden; 2 Meetme Psykologkonsult AB, Lerum, Sweden; 3 Department of Clinical Neuroscience, Center for Psychiatry Research, Karolinska Institutet, Stockholm, Sweden; 4 MicLab AB, Stockholm, Sweden

**Keywords:** Inspections, enforcement, motivational interviewing, compliance, waste separation behaviour, field experiment

## Abstract

This field experiment investigates the effect of first-time inspections of restaurants’ waste sorting and explores whether motivational interviewing (MI) training of inspectors in this specific setting enhances the propensity of restaurants to be compliant with regulations. Our results show strong positive effects of first inspections with an average improvement of 55%. Also, the MI training of inspectors seems to affect compliance. However, this may also be a combined effect of the first inspection, MI training and more days between inspections. Further research is needed.

## Introduction

World economic growth in the last two centuries has produced a dramatic increase in municipal solid waste (MSW) generation (Calabrò and Satira, 2020). Not addressing this challenge efficiently will lead to environmental and health costs as well as lost economic opportunities from recycling of waste. An efficient MSW management system is therefore vital for tackling this challenge. Recent research has shown that a management system with separate collection of MSW delivers superior results (Calabrò and Satira, 2020). Such a system ‘practically aims to optimize the management of each waste component (e.g. organics, paper, glass, metals, mixed waste) maximizing recovery (i.e. materials and energy) and minimizing landfilling and environmental impacts’ (Calabrò and Komilis, 2019, p. 184). Still, for the system to work as intended, the individuals and/or organizations that generate the waste must be sufficiently educated and incentivized to sort the waste properly and sustainably (Calabrò and Satira, 2020).

This study focuses on restaurants which generate a mix of different waste fractions: food, different packaging materials such as plastics, paper, tin, etc. In Sweden, the Environmental Code, in accordance with EU directives, regulates that restaurants must sort their waste with the ambition of advancing the restaurants’ waste behaviour. However, unless backed up by inspections and enforcement, the environmental legislation may only be interpreted as guidelines leading to little actual sorting (Gray and Shimshack, 2011).

In this study, we explore how restaurants’ waste sorting behaviour can be improved by inspections of their waste sorting procedures. We also investigate how the communicative method ‘motivational interviewing’ (MI) can be adapted to the context of restaurant inspections and whether MI skills applied in inspections can improve compliance with legislation.

The study was carried out as part of the interdisciplinary research programme, Inspections and Enforcement as Instruments for Enhancing Environmental Behaviour (‘Tillsynen som styrmedel för ett förbättrat miljöbeteende’ – TSFM). An earlier, and longer, version of this paper is contained in the final report of the TSFM research programme (Chapter 5 in Herzing and Jacobsson, 2017). This study was designed as part of a project in five Swedish municipalities, with the aim of inducing restaurants to sort their waste in accordance with the law. These five municipalities – Falköping, Hjo, Karlsborg, Skövde and Tibro – coordinate inspections and enforcement of environmental legislation within the regional collaboration Miljösamverkan Östra Skaraborg (MÖS). The project involved seven food safety inspectors who regularly carry out inspections of restaurants to check compliance with food safety legislation. These inspectors had noted that many restaurants do not sort their waste, which is regulated by the Environmental Code’s Ordinance on waste management. Since restaurants are not inspected by environmental inspectors, no active efforts had previously been undertaken to monitor and improve restaurants’ waste sorting. MÖS therefore decided to add waste management to the food safety inspectors’ checklist for restaurants and to actively motivate restaurants to sort waste as required by legislation. The fact that restaurants’ waste sorting had previously not been monitored by inspectors provided a unique opportunity to design a research study analysing how inspections per se and adding MI skills affect compliance with legislation. It is generally very hard to find situations where inspections are carried out for the first time and thus equally difficult to evaluate the effect of inspections with the counterfactual of no inspections as opposed to the counterfactual of less frequent ones. Also, adding skills in an evidence-based communication methodology to the inspector toolbox made the study even more interesting.

The seven MÖS inspectors were trained in MI, an evidence-based method to change behaviour through communication. MI can briefly be defined as ‘a collaborative conversation style for strengthening a person’s own motivation and commitment to change’ (Miller and Rollnick, 2013). The presentation of MI and the training programme as well as the methodological approach is similar to our parallel study (Wickström et al., 2017). MI is based on the assumption that people prefer to take their own decisions regarding matters that affect them and that they may take offence when their choices are questioned. MI counsellors are trained to interact in an empathic and collaborative manner with clients. Information should be integrated in and adapted to the dialogue with the client to make it more likely to be accepted and understood. MI also requires counsellors to evoke a client’s change talk. Several studies (Apodaca and Longabaugh, 2009; Gaume et al., 2013; Lindqvist et al., 2017) have shown correlations between clients change talk (e.g. expressing reasons in favour of or against change) during conversations and the realization of actual behaviour. Change talk is also about expressing beliefs in the ability to change undesirable behaviours and to decrease talk about the inability to change.

MI has been widely used for treating health behaviour problems and has a solid research based on randomized controlled studies during nearly 40 years, which have mainly shown significant low to moderate effects with respect to, for example, reducing or stopping drinking problem (Lundahl et al., 2010), stopping the use of illegal drugs and tobacco (Lundahl et al., 2013) and completing a treatment programme (Hettema et al., 2005). The positive effects of MI have contributed to its dissemination within health care, but rarely beyond this context, with a few exceptions. For example, Thevos et al. (2000) demonstrate how MI can enhance the adoption of clean drinking water practices. Moreover, Klonek and Kauffeld (2012) show that MI can be used in conversations with people about their environmental behaviour and that MI increases pro-environmental verbal behaviours compared to controls. In addition, it has been shown that MI significantly improved veterinarians’ communication skills in veterinary heard health management (Svensson et al., 2020). In a previous research programme, Efficient Environmental Inspections and Enforcement (‘Effektiv miljötillsyn’ – EMT), our research team carried out a MI training programme aimed at environmental, food safety and health safety inspectors, who experienced the MI training as beneficial in their work (Forsberg et al., 2014, 2016). To our knowledge, the EMT research programme was the first to adapt MI to an inspection setting. In a parallel study, we further developed the MI training programme to explore the applicability of MI for inducing property owners to carry out radon gas radiation measurements (Wickström et al., 2017). Our hypothesis was that MI may contribute to more effective work regarding environmental issues. We expected MI to be useful in evoking reasons in favour of environmentally responsible behaviour, for example, a desire to preserve the environment for one‘s children and grandchildren, which might counteract the incentives to minimize short-term consequences (e.g. economic costs).

Results from the earlier EMT study showed that after having completed a MI training programme, inspectors significantly improved their skills in conversations with inspectees regarding two important MI variables, showing empathy and evocation. The average empathy score changed from 1.36 to 1.79 and the average evocation score changed from 1.3 to 1.83 on a Likert scale ranging from 1 (‘low’) to 5 (‘high’) (Forsberg et al., 2014, 2016). The inspectors also experienced MI to be of benefit during inspections and became more satisfied with their own performance at inspections. However, the EMT MI training programme covered a wide range of inspection settings and was devised for heterogeneous groups of inspectors working in different contexts, which made it difficult to convey the applicability of MI. In contrast to the EMT research programme, this study has been adapted to a specific setting, allowing the participants to be focused on the communicative requirements in their context. Hence, we expected inspectors to attain a higher MI competence than in the previous EMT training programme. Results from the parallel study (Wickström et al., 2017) covering inspectors making phone calls to property owners to induce the measuring and reporting of radon gas radiation confirm this expectation. For example, the inspectors’ average empathy score changed from 1.78 to 3.35 and the evocation score changed from 1.65 to 3.03 on a Likert scale ranging from 1 (‘low’) to 5 (‘high’).

The aim of this study is to investigate the effectiveness of inspections on restaurants’ compliance with waste sorting legislation and to explore the effects of MI training of inspectors in this specific setting. The intention is to analyse how restaurants’ compliance with regulations is affected by previous inspections (an inspection effect) and by the MI training programme (a combined inspection and MI effect). The study was approved by the Regional Ethical Review Board in Stockholm (reg nr 2014/1417-32).

## Materials and methods

### Research design

Our study was designed to enable an evaluation of how inspections and MI training of inspectors affected the restaurants’ rate of compliance with waste sorting regulations. Using a field experimental setup, we contribute towards the empirical testing of new environmental policy measures. See, for example, List and Price (2016) for a discussion on the use of field experiments in environmental and resource economics.

In 2014, MÖS determined that regular food safety inspections of restaurants should also involve controlling compliance with waste sorting regulations. Our study covers the first four inspection campaigns, called sprints, where restaurants’ waste sorting was inspected. A sprint generally lasts for about 1 month. During the sprints of this project, some inspectors conducted inspections of pre-determined groups of restaurants. These four sprints took place between the Autumn of 2014 and the Autumn of 2015. Between the first and the second sprints, all seven food safety inspectors participated in a MI training programme, which consisted of five training days spread out over a 3-month period.

The study covered 181 registered restaurants. A total number of 359 inspections were carried out in the four sprints. One of the inspectors dropped out of the study during the MI training programme for health reasons. Table 1 shows the schedule of the study and provides information on the number of restaurants being inspected and the number of inspectors participating in each sprint.

**Table 1.** Schedule of the study. Not all inspections in sprint 3 could be carried out within 1 month, and hence had to be postponed to the summer when one of the inspectors inspected the remaining restaurants.

Date	Activity
29 September–24 October 2014	Sprint 1: 62 inspections, 7 inspectors
November 2014–January 2015	MI TRAINING PROGRAMME
10 February–6 March 2015	Sprint 2: 116 inspections, 5 inspectors
20 April–7 August 2015	Sprint 3: 46 inspections, 3 inspectors
August 2015	Extra MI training
28 September–12 November 2015	Sprint 4: 135 inspections, 6 inspectors

MI: motivational interviewing.

Restaurants were randomly assigned to inspectors as well as the order in which restaurants were inspected. At inspections, inspectors made an ocular assessment to determine whether restaurants comply with waste sorting regulations such that we obtained a binary outcome variable (to keep things simple, the degree of non-compliance was not accounted for). In normal routine, non-compliance leads to an injunction prescribing the inspectee to undertake measures to achieve adherence. However, in this research project, violations of waste sorting regulations were not followed by an injunction – the purpose was to avoid distortions in the analysis of the impact of inspections and MI on compliance.

To evaluate how inspections and the MI training programme affected compliance, we had to observe changes in compliance over time, and hence 49 restaurants that were inspected only once had to be excluded. Thus, the number of observations was reduced from 359 to 310, corresponding to a decrease in the number of included restaurants from 181 to 132. There were 40 restaurants that were inspected more than twice, of which six were inspected four times (i.e. in every sprint). However, this study focuses only on the two first inspections for each restaurant. Hence, if an inspector at the first inspection succeeded in motivating the restaurant to comply with waste sorting regulations, this became apparent at the second inspection of that restaurant. We were able to obtain measures for both a pure inspection effect and a combined inspection and MI effect.

For the first group of restaurants, the control group coded as 0, the first inspection took place during the first sprint before inspectors had received any MI training, that is, they were inspected by a MI-untrained inspector. By comparing the outcomes at the two first inspections among these restaurants, we could identify a pure inspection effect. Any change in compliance in this group is thus likely to be attributed to waste sorting having been inspected for the first time.

The second group of restaurants, the treatment group coded as 1, was inspected for the first time after inspectors had completed the MI training programme and was then inspected again. Since MI-trained inspectors carried out the first inspection, the difference in outcomes between the first two inspections is thus likely to be attributed to both an inspection and a MI interaction effect.

Food safety inspections are by law supposed to be conducted without prior notification. However, sometimes inspections are notified in advance for practical reasons, for example, if the restaurant has irregular opening hours or if it has been closed at several unannounced inspector visits. In our sample, there is a total number of 24 inspections where restaurants were notified in advance and coded as 1 else 0; of these 5 were used to evaluate changes in compliance.

### The MI training programme

Six food safety inspectors participated in the study, five women and one man between the ages of 23 and 57 years. They all had university education in natural sciences. The inspectors had 2–13 years of inspection and enforcement work experience, and all of them had conducted food safety inspections during the last 12 months. None had previous knowledge of MI, but all had received some training in communication skills (Table 2).

**Table 2.** Descriptive statistics of the participating inspectors.

Number in study	Sex	Age	Number of years of education	Years of experience of enforcement
7	Female	37	15	13
5	Male	25	15	2
3	Female	40	15	9
4	Female	44	15	10
2	Female	59	15	2
1	Female	56	15	5

The inspectors received 30 hours of training during 5 days spread out over a 16-week period. Each training day consisted of short theoretical introductions mixed with exercises of MI skills. During the last 3 hours of all training days (except the first one), the inspectors gave each other feedback to recorded conversations under the supervision of the instructor. Before training days 3 and 5, each participant recorded an inspection conversation about waste sorting, which was uploaded to a homepage to be professionally coded for use as feedback by the MI instructor. The MI coding was made according to the Motivational Interviewing Treatment Integrity manual by MIC Lab. The coding of MI at MIC Lab is validated, telling ‘How much MI is it in this conversation?’ (Moyers et al., 2014, 2016).

The choices of MI themes for each training day were made according to common MI practice (Miller and Moyers, 2007; Miller and Rollnick, 2013) and built on our experiences from the MI training programmes of EMT (Forsberg et al., 2014, 2016) as well as our recent study on the application of MI to induce radon gas radiation measurements by property owners (Wickström et al., 2017). In addition, the training programme also included a brief introduction to fundamental reinforcement principles within psychological learning theory (Sundel and Sundel, 2005).

### Statistical analyses

All analyses were made in SPSS 26.0 and STATA 13 software. Comparison between groups was made using Mann–Whitney U-test. The association between compliance at follow-up and the MI group, controlling for initial compliance at baseline, notification of inspection and time period between inspections, was evaluated using multi-variable regression analyses.

## Results

The control and treatment groups are described in Table 3 in number of inspections, average on compliance with waste sorting regulations (0 = no waste sorting and 1 = waste sorting) at inspection 1, average number of days between inspection 1 and 2 and notified at inspection 2.

**Table 3.** Descriptive statistics of the control and treatment groups.

	Control group	Treatment group
*N*	52	80
Compliance rate inspection 1	0.385	0.338
Average number of days between inspections	171	228
Notified before inspection 2	2	3

We can note from Table 3 that the treatment group has (i) more restaurants, (ii) a lower degree of compliance at inspection 1, (iii) on average 57 days longer time period between the inspections and (iv) one more restaurant that was notified before inspection 2 than the control group. We did not succeed in achieving similar characteristics because the quasi-experimental design also had to fit into the daily operations of the inspection authority. We will return to how this might affect the results.

Table 4 provides average compliance rates for restaurants in the first and second inspection for both the control and treatment groups.

**Table 4.** Number of restaurants and average compliance rates for control and treatment groups. The last column reports the *p* value from a two-sample test of proportions for the alternative hypothesis that the difference is equal to 0, that is, there is no difference between the average compliance rates of inspection one and two.

	*N*	Insp. 1	Insp. 2	Diff	*p* Value
TOTAL	132	0.356	0.553	0.197	0.001
Control group	52	0.385	0.462	0.077	0.427
Treatment group	80	0.338	0.613	0.275	0.001

Table 4 indicates an increase in average total compliance rate between the first and the second inspection from 0.356 to 0.553, which represents an increase of 55% that is also statistically significant. This improvement in the compliance rate is stronger in the treatment group. The average compliance rate in the control group increased by 7.7 percentage points from 0.385 to 0.462 (a 20% increase). This improvement in compliance can be seen as a pure inspection effect. However, this effect is not statistically significant. It is hard to know whether this is due to the smaller group size (*N* = 52), the lack of MI training, the relatively high degree of initial compliance (we find that previous compliance behaviour is a strong predictor for future behaviour), or a combination of these factors.

Among the 80 restaurants in the treatment group, the average compliance rate rose by 0.275 percentage points from 0.338 to 0.613 (a 85% increase). In this group, the improvement in compliance can be attributed to having been inspected by a MI-trained inspector at the first inspection. Hence, besides the inspection effect, a MI effect is also present. The combined effect of a first inspection and a MI-trained inspector amounts is, in contrast to the pure inspection effect, strongly statistically significant.

Hence, our results, as presented in Table 4, suggest strong effects from a combination of inspections and MI training on restaurants’ compliance. Let us now perform a more nuanced analysis of these effects by means of regression analysis.

A number of factors may affect the behaviour of the restaurants. Firstly, as previous behaviour is generally a good predictor for future behaviour, the probability of being compliant in inspection 2 is likely to be affected by past behaviour, that is, compliance in inspection 1. Also, some restaurants were, for practical scheduling reasons, notified about the second inspection which, in turn, may affect behaviour. However, in the notification, there was no mention about specific controls for waste sorting. Also, as the time between the first and second inspection varied, as outlined in Table 3, the effects of the first inspection may either be amplified or reduced by a longer interval. If the interval between the inspections is very short, any changes in restaurant behaviour as a result of the first inspection may not have had sufficient time to be implemented. On the other hand, if the interval is very long, the effects of the first inspection might wear off. For these reasons, we introduce the following variables in our regression analysis.

Compliant_2_i_ Restaurant i was found to be compliant during inspection 2. This is a dummy variable with value=1 if in compliance, 0 otherwise.

Compliant_1_i_ Restaurant i was found to be compliant during inspection 1. This is a dummy variable with value=1 if in compliance, 0 otherwise.

MI_i_ Restaurant i was inspected by a MI-trained inspector during inspection 1. This is a dummy variable with value=1 if the inspector was MI-trained, 0 otherwise.

Notified_2_i_ Restaurant i was notified in advance of inspection 2. This is a dummy variable with value=1 if the restaurant was notified, 0 otherwise.

Dist_i_ The number of days between the inspections.

Table 5 has some descriptive statistics of the main variables.

**Table 5.** Descriptive statistics of the regression variables.

Variable	Obs	Mean	Std dev	Min	Max
Compliant_1	132	0.356	0.481	0	1
Compliant_2	132	0.553	0.499	0	1
MI	132	0.606	0.490	0	1
Notified_2	132	0.038	0.192	0	1
Dist	131	205	54	64	379

We set up the following linear ordinary least squares (OLS) regression model in equation (1):

(1) $$\begin{array}{c} \mathrm{Compliant}\_2_{i}=\beta_{0}+\beta_{1}{\mathrm{MI}}_{i}+\beta_{2}\mathrm{Compliant}\_1_{i}\\ +\beta_{3}\mathrm{Notified}\_2_{i}+\beta_{4}{\mathrm{Dist}}_{i}+u_{i} \end{array}$$

Column (1) in Table 6 has the basic regression, while columns (2), (3) and (4) introduce controls for compliance in inspection 1, prior notification on inspection 2 and finally the number of days between inspections.

**Table 6.** Regression results.

Dependent variable Compliant_2	(1)	(2)	(3)	(4)
MI	0.15* (0.089)	0.16* (0.086)	0.16* (0.086)	0.13 (0.099)
Compliant_1		0.27*** (0.084)	0.27*** (0.084)	0.26*** (0.087)
Notified_2			−0.17 (0.224)	−0.16 (0.219)
Dist				0.0005 (0.0009)
Intercept	0.46*** (0.070)	0.36*** (0.075)	0.36*** (0.076)	0.28 (0.170)
Robust standard errors	Yes	Yes	Yes	Yes
*F*-test				
Model	2.90 (0.09)	7.56 (0.001)	5.28 (0.002)	3.91 (0.005)
Observations	132	132	132	131
*R* ^2^	0.020	0.09	0.095	0.098

*p* Values are reported in brackets below the *F*-values.

MI: motivational interviewing.

Significance levels for coefficients: *p < 0.10. **p < 0.05. ***p < 0.01.

Qualitatively similar results were found when running logit regression on the same variables. However, we chose to represent the results using the linear OLS regressions as the coefficients and then receive a more straightforward interpretation.

Model (1) mirrors Table 4 as the intercept equals 0.46, denoting the probability that a restaurant in the control group is in compliance at inspection 2. This indicates, as mentioned before, an average improvement of 7.7 percentage points in comparison to inspection 1; however, this difference is not statistically significant (see Table 3). Also, the coefficient for MI equals 0.15 meaning that the average probability of a restaurant in the treatment group to be compliant is 15 percentage points higher than a restaurant in the control group in inspection 2. This difference is significant at the 10% level.

Model (2) controls for the compliance of restaurants at inspection 1 and finds that this variable is very important, and statistically significant at the 1% level, in predicting the outcome in inspection 2. A restaurant that was compliant in inspection 1 is, on average, 27 percentage points more likely to be in compliance in inspection 2 compared to a restaurant that was not compliant in inspection 1.

Model (3) confirms the results of model (2) when controlling for prior notification which does not appear to have any statistically significant effect. Model (4) controls for the number of days between inspections. We can see that all coefficients then turn insignificant except for the coefficient for previous compliance, $\beta_{2}$ , which remains very stable. The coefficient for MI is reduced to 0.13 and now has a *p* value of 0.15. This may be a consequence of the treatment group having, on average, 57 more days between inspections than the control group (see Table 2). Also, the coefficient for the number of days between inspections does not have a (statistically significant) effect on behaviour at inspection 2.

## Discussion

We can thus not claim, with statistical significance, that we have confirmed the hypothesis and identified that MI training in combination with an inspection has an effect when controlling for the number of days between inspections. Is it in fact the longer time between inspections that drives the positive MI effect? An argument in support of this hypothesis is that more time between inspections increases the possibilities for restaurants to make changes that would support compliance like introducing new routines, purchasing new equipment, etc. On the other hand, a longer time between inspections might decrease the effect of inspection as the initial effect wears off.

Given the relatively small number of observations, it is also possible that the difference in days between inspections for the control and treatment group simply causes statistical noise in the regression analysis, thus leading to the loss of significance.

The regression analysis found that previous behaviour is a very good predictor for future behaviour. Also, the addition of MI training appears to have a positive effect on the restaurants’ compliance, but future research has to control for the number of days in between inspections.

Almost 2 years after the last inspections in this study, a follow-up study was conducted (Herzing and Wickström, 2019). This study found that average compliance had decreased among the restaurants that participated in our study since their last inspection. The average decrease in compliance varied across groups of restaurants depending on how many inspections they had received in the first study: 3.7 percentage points for restaurants which only received one inspection, 7.5 percentage points for restaurants which received two inspections and 3.5 percentage points for restaurants which received three inspections. The compliance rates for restaurants that received two or three inspections were, however, higher in the follow-up inspection than in their very first inspection, indicating that repeated inspections provide a long-run positive, albeit depreciating, compliance effect.

## Conclusion

The aim of this study was to investigate the effectiveness of inspections on restaurants’ compliance with waste sorting legislation and to explore the effect of MI training of inspectors in this specific setting. Our main conclusions are the following.

The compliance rate of the 132 restaurants rose from 0.36 in the first inspection to 0.55 in the second inspection – an average improvement of 55%. A big and statistically significant effect. Hence, it appears that inspections with a component of MI training (in our case 61% of restaurants received MI-enhanced inspections) has a positive effect on compliance.

We also found that the average improvement in the control group was 7.7 percentage points and not statistically significant. This would correspond to the pure inspection effect. On the other hand, we found a statistically significant improvement of 27 percentage points in the treatment group. This finding would suggest that MI training is important for inspections to have a positive effect on compliance.

When conducting a more nuanced multivariate regression analysis, we found that compliance behaviour in inspection 1 was a good predictor of behaviour in inspection 2. Also, the coefficient of the MI variable was found to be positive, as expected, but when we controlled for the number of days between inspections, we lost the statistical significance of this coefficient. Hence, even though we know that the treatment group’s larger improvement in compliance, compared to the control group, was both substantial and significant, we cannot say, with statistical certainty, what factor drove this result. We cannot be sure whether the improvement is due to the MI training, the extra number of days between the inspections or a combination of both. We leave to future research to find out.

In sum, our results imply that inspections matter for compliance and that it is probably a good idea to train inspectors in MI techniques.
